# Supplementary material for: From Clinic to Reality: Integrating Sound Field Testing and Hearing Quality Measures in Cochlear Implant Users
Source: J Clin Med. 2025 Nov 27;14(23):8430. doi: 10.3390/jcm14238430 (PMC12693144; doi:10.3390/jcm14238430)
Supplement: Supplementary file 1 [file jcm-14-08430-s001.zip › jcm-3933602-supplementary.pdf]

Journal of
Clinical Medicine

Article

From Clinic to Reality: Integrating Sound Field Testing and Hearing Quality Measures in Cochlear Implant Users

Marta Álvarez-Cendrero [1,*], Manuel Lazo-Maestre [2], Serafín Sánchez-Gómez [2] and María A. Callejón-Leblic [2]

[1] Department of Otolaryngology, Head and Neck Surgery, De La Merced University Hospital, 41640 Osuna, Spain; malvarezc@euosuna.org
[2] Department of Otolaryngology, Head and Neck Surgery, Virgen Macarena University Hospital, 41009 Seville, Spain; manuel.lazo@faigesco.es (M.L.-M.); mcallejon@us.es (M.A.C.-L.)
* Correspondence: malvarezc@euosuna.org

Supplementary Material

Data and outcome measures

We want to extend our data analysis for the reader to gain context, to further explain and generalize or restrict our results.

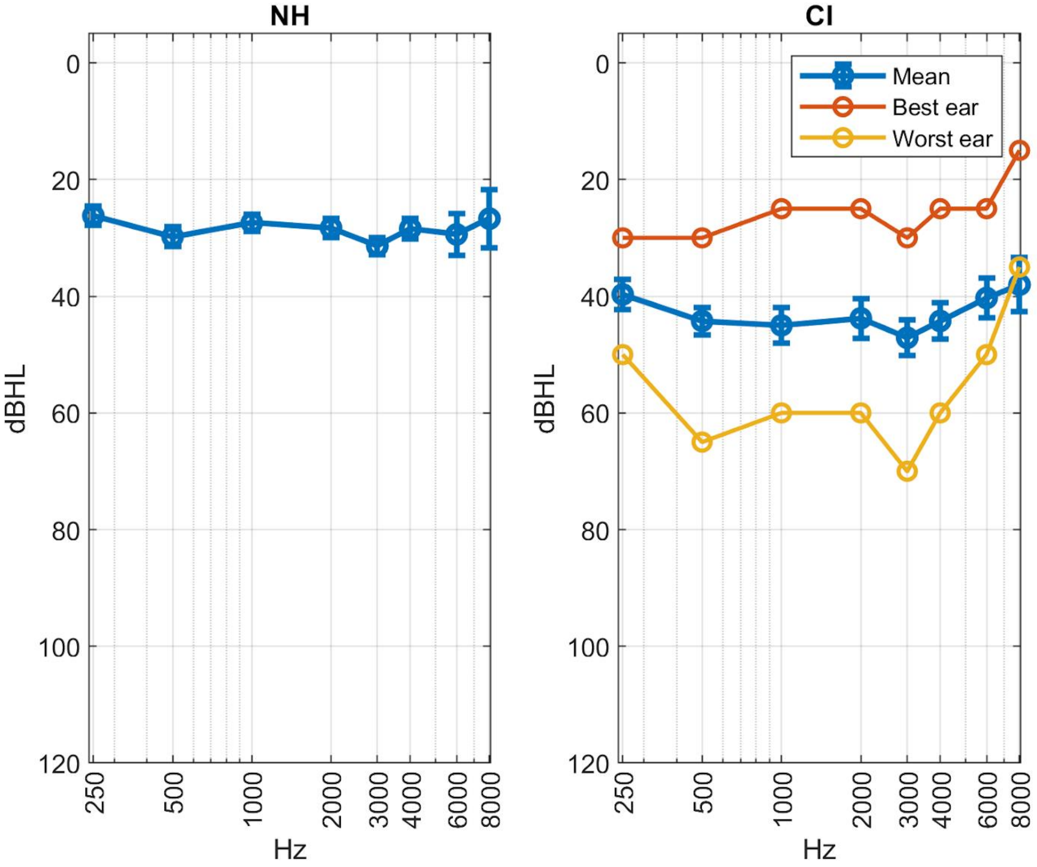

Figure S1. Dual plot of the mean PTA values from the main groups, NH and CI users.

Figure S1 shows a dual plot of the main groups and its mean audiometry values. We inspected the best and worst ear for CI users, and it is included in the figure as a way to notice the wide range in which CI users results spread. Clearly both groups are separated

 https://doi.org/10.3390/jcm14238430

by about 15-20 dBHL but using only mean values have no classifying impact when considering a single individual as there is a big variability.

As we can see in the Figure S2, NH is also very noisy specially in the high frequency domain, where they show more variability than CI users.

Correlation Analysis

In addition to the material showed in the main section of the paper, we include this Table S1 with the spearman correlation coefficients but obtained for the overall sample, the NH and the CI. This table is related to Table 2 and Table 3.

Table S1. Spearman correlation coefficients for overall sample. *p- value < 0.05; ** p < 0.01; *** p < 0.001.

	**Age**	**PTA**	**WRS quiet**	**WRS sea**	**WRS traffic**	**WRS cafe**	**Sound localization**	**SSQ12**
**Age**	1	0.575***	-0.218*	-0.304**	-0.424***	-0.491***	-0.303**	-0.448***
**PTA**	0.575***	1	-0.678***	-0.737***	-0.813***	-0.870***	-0.656***	-0.638***
**WRS quiet**	-0.218*	-0.678***	1	0.707***	0.709***	0.668***	0.670***	0.514***
**WRS sea**	-0.304***	-0.737***	0.707***	1	0.887***	0.806***	0.493***	0.504***
**WRS traffic**	-0.424***	-0.813***	0.709***	0.887***	1	0.912***	0.580***	0.642***
**WRS cafe**	-0.491***	-0.870***	0.668***	0.806***	0.912***	1	0.625***	0.664***
**Sound localization**	-0.303**	-0.656***	0.670***	0.493***	0.580***	0.625***	1	0.536***
**SSQ12**	-0.448***	-0.638***	0.514***	0.504***	0.642***	0.664***	0.536***	1

We can see that the most correlated variables are the WRS tests and as explored previously, there is still a mild relation between PTA and WRS tests. As could be expected, there is a less relevant correlation between PTA and age than in the table with NH (Table 2) but greater than the value obtained in the CI case (Table 3).

Regression analysis

In relation to linear regression models applied to the complete sample (Table S2), Age, Sex, PTA, WRS_Quiet, WRS_Sea, WRS_Traffic, WRS_Cafeteria; Localization were considered as independent variables, whereas we designated as dependent variables: Age [SSQ12 (-2.240 ± 1.022), [-4.275, -0.205], (p= 0.031)], (R2= 0.538); PTA [Age (0.082 ± 0.038), [0.006, 0.158], (p= 0.036); WRS_Cafeteria (-0.209 ± 0.025), [-0.259, -0.158], (p < 0.001)], (R2= 0.771); WRS_Cafeteria [PTA (-2.400 ± 0.23), [-2.873, -1.927], (p < 0.001); SSQ12 (3.234 ± 1.094), [1.056, 5.412], (p= 0.004)],(R2= 0.768); WRS_noise [PTA (-0.146 ± 0.018), [-0.182, -0.109], (p< 0.001)], (R2= 0.436); SSQ12 [Age (-0.025 ± 0.011), [0.048, -0.002], (p= 0.037)], WRS_Cafeteria [(0.04 ± 0.005), [0.029, 0.052], (p< 0.001)], (R2= 0.502); Localization [PTA (-1.667 ± 0.232), [-2.129, -1.206], (p< 0.001)], (R2= 0.502).

Table S2. Multivariant analysis for CI+NH group (overall sample). Independent Variables: Age, Sex, PTA, WRS_quiet, WRS_sea, WRS_traffic, WRS_cafe, Localization

	**Adjusted b (mean ± SE)**	**95% CI**	**p - value**	**R2**
**Age**				0.538
SSQ12	-2.240 ± 1.022 a	[-4.275, -0.205]	0.031	
**PTA**				0.771
Age	0.082 ± 0.038 b	[0.006, 0.158]	0.036	
WRS cafeteria	-0.209 ± 0.025 b	[-0.259, -0.158]	< 0.001	
**WRS cafeteria**				0.768
PTA	-2.400 ± 0.238c	[-2.873, -1.927]	< 0.001	
SSQ12	3.234 ± 1.094 c	[1.056, 5.412]	0.004	
**WRS quiet**				0.489
PTA	-0.728 ± 0.0822	-0.8927, -0.5657	<0.001	
**WRS noise**				0.436
PTA	-0.146 ± 0.0183	-0.182, -0.109	<0.001	
**SSQ12**				0.502
Age	-0.025 ± 0.011	-0.048, -0.002	0.037	
WRS cafeteria	0.04 ± 0.005	0.029, 0.052	<0.001	
**Localization**				0.386
PTA	-1.667 ± 0.232	-2.129, -1.206	<0.001	

Age groups

In relation to the age - categorized groups (Table 6), the mean age of NH was 27.7 ± 4.9 [20 – 40] in group 1, 48.0 ± 6.9 [41 – 58] in group 2 and 63.1 ± 3.2 [60 – 73] in group 3. The proportion of female participants in these groups was 64.7%, 62.5% and, 70.6% respectively. On the other hand, in the CI group, the mean age was 35.0 ± 4.3 [26 – 40] in group 1, 50 ± 8.2 [41 – 59] in group 2 and 69.3 ± 6.3 [61 – 79] in group 3. The proportion of female participants in each group was 71.4%, 70% and 52.9%, respectively.

A statistically significant difference in PTA (pure tone audiometry) was observed across age groups in the NH group (U= 69.50; p< 0.01), as well as a strong correlation between age and PTA (r= 0.748; p< 0.01).

Additionally, a statistically significant difference was found in hearing thresholds from 500 Hz onwards (Table 7), with the difference becoming more pronounced as frequency increased in the NH group (Table 7). No statistically significant difference was observed in the CI group.

Age was associated with worse speech recognition (Rho = -0.61; p < 0.01), and worse localization ability (Rho = -0.039 ; p < 0.01) in the NH group. Such a difference was greater in the younger age group from 21 to 41 years, decreasing as age increases (Figure 1).

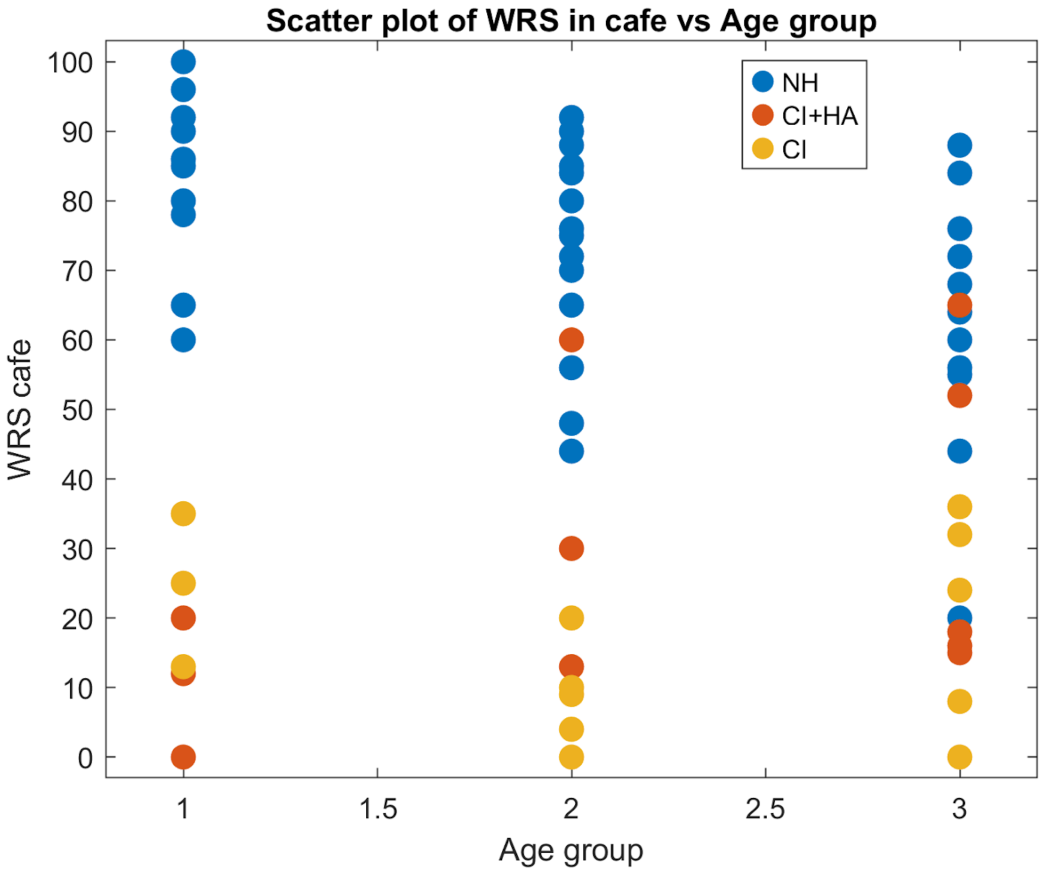

Figure S2. Boxplot representation of the age groups: 18-40, 41-60 and +60 years old. The figure also indicates the WRS cafe scenario and each datapoint is color-coded with the auditory group.

Both auditory abilities, speech recognition and sound localization ability are correlated (R2= 0.4; p< 0.01).

Additionally, higher scores on the SSQ-12 questionnaire were associated in both groups with better intelligibility (r = 0.70; p < 0.01), improved sound localization (r = 0.54; p < 0.01), and lower auditory thresholds (r = 0.47; p < 0.01).

Table S3. Distribution of demographic characteristics according to age ranges.

	Group 1 (n = 24)	Group 2 (n = 26)	Group 3 (n = 34)
$\bar{x}$ age ± SE	29.8 ± 7.6 [20 – 40]	48.7 ± 7.3 [41 – 59]	67.7 ± 4.9 [60 – 79]
NH	27.7 ± 4.9 [20 – 40]	48.0 ± 6.9 [41 – 58]	63.1 ± 3.2 [60 – 73]
CI	35.0 ± 4.3 [26 – 40]	50 ± 8.2 [41 – 59]	69.3 ± 6.3 [61 – 79]

Table S4. Mean thresholds according to age ranges in NH group.

	t- Student	95% CI	p - value
250 Hz	-3.881	[-10.772 – -3.346]	< 0.001
500 Hz	- 4.380	[- 12.066 – -4.405]	< 0.001
1 K Hz	-6.000	[-10.637 – -5.245]	< 0.001
2 K Hz	-6.456	[-12.768 – -6.644]	< 0.001
4K Hz	-7.040	[-14.031 – -7.734]	< 0.001
8 KHz	-6.700	[-39.120 – -20.880]	< 0.001

MANOVA analysis

The following Figure S4 shows the group division that MANOVA was able to perform. There is a clear difference between NH and CI users, but no canonical space was found that could divide CI+HA from CI.

.

As stated in the main text, the correlation between the canonical variables and the independent variables was calculated and it is represented in Figure S5.

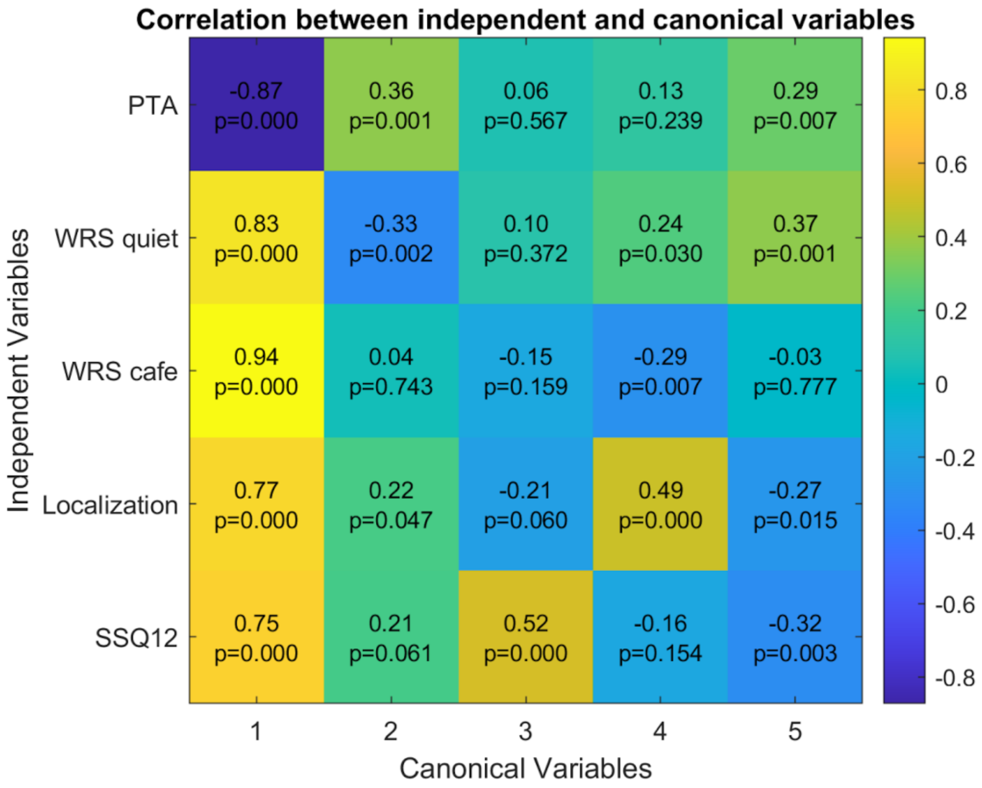

Figure S3. Correlation matrix between CV and independent variables.

We then went ahead and generated a combined plot with the data in a scatter plot represented in a base formed by the two main CVs and then we added as vectors the independent variables correlation with those CVs. See Figure S6

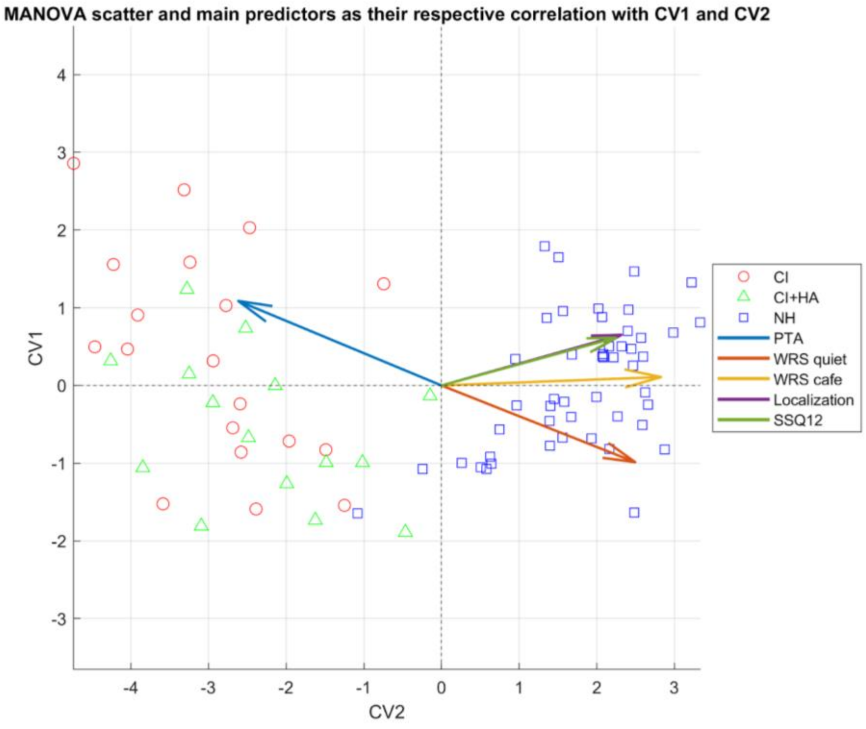

Figure S4. Scatter plot of the most relevant CVs on the axes after MANOVA. The key predictors are also displayed according to their correlation with the CVs but scaled to facilitate inspection.

This way we were able to view all the previous information and identify correlation between independent variables. In this case SSQ12 and Localization seemed to be highly correlated in the solution space created by the CVs.

With this we expected to be able to improve the explainability of the results. As stated in the main sections of the article, the model considers as relevant all the independent variables as no one of them is understated by the CVs. But we are interested in using CVs to separate in the main groups of interest, so we searched for combinations of CVs that were able to help differentiate CI from CI+HA users. Unfortunately, we did not find a combination of CVs that clearly provides that separation. As stated previously, perhaps a greater amount of data or maybe including other variables from each subject could help with this task.

Machine learning analysis

Machine learning enables algorithms to learn directly from data and build statistical representations and classifiers, among other capabilities. In audiology, there is a common situation when multiple sources of data have to be considered simultaneously to fully comprehend the phenomena underlying a certain condition. This approach resolves some limitations of other methods e.g. the complexity of searching for non-linear relations.

In order to improve our understanding of the relationships between the variables, a series of trials were conducted with the use of machine learning algorithms. We trained a wide range of classification and regression models to abstract the results from the peculiarities of each architecture. We tried to predict: PTA, WRS in all situations, Age and Hearing Group using different subsets of the data. We then analyzed the results to find the most valuable predictors in each case as we are interested in the explainability of the results more than in generating optimal models because our dataset is not big enough for that matter.

In each case we used the Classification and Regression learner apps of MatLab. This allowed for easy comparison of many architectures and models from the following families: decision trees, discriminant analysis, logistic regression and logistic regression classifiers, naive bayes classifiers, SVMs, linear classifiers, KNN, neural networks and ensembles among others.

In these experiments, the key variable to be classified is the age group. First a classification session was conducted trying to predict the age group and using all the available data except for the age were every architecture and model presented poor results. We then removed the audiometry data and observed that all models showed poorer performance. Up to this point, the data regarding CI details were still being fed into the algorithms. For the NH group, those variables were treated as missing values. When a third session was conducted with all those variables removed, the models once again showed poorer performance. Then, data from CI and NH groups were divided to train models separately. In the CI group, a general but mild improvement was observed, with a slight decrease in a few models. In the NH group a general and moderate improvement was noted. Finally the best models in each session were inspected to analyze the key predictors: for the audiometry data, we found that in some models, specific thresholds -with a mild tendency towards high frequency in standard audiometry, like 6k and 8k Hz- for key frequencies could be more important than PTA4; in the CI group, considerable inconsistency was observed in the analysis of key predictors, with nearly all top models producing divergent results. The other classification session conducted used the hearing group instead of age and yielded the best results. The most common predictors among the top models were:

WRS_quiet, WRS_cafe, audiometry threshold for 250 Hz, WRS_traffic, PTA4 and SSQ12 questions.

For the regression tasks we tried to predict WRS in different situations and PTA4 but only used SSQ12 results for each question or SSQ12+Age as input data. The most relevant results are explained below:

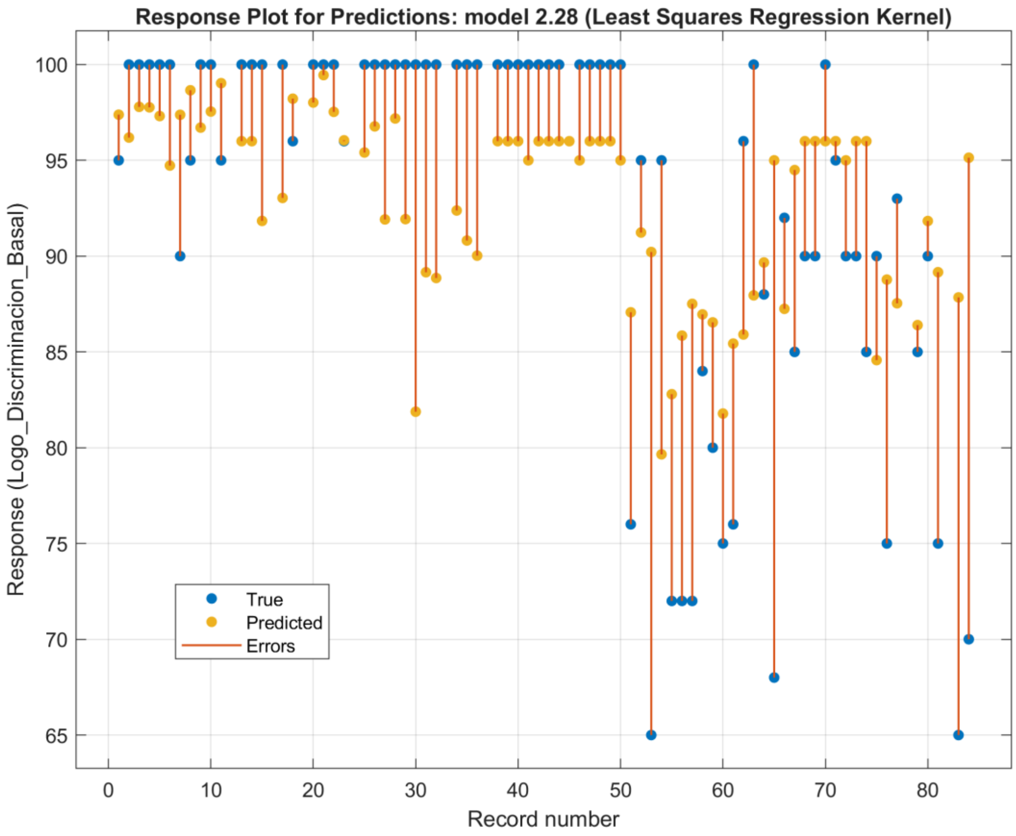

Figure S5. Response plot for model 28 (Kernel type). Original data is shown as blue dots, predicted data as yellow dots and RMSE as the orange lines connecting both. The first 50 observations are the NH and the last ones the CI users. Data was randomly fed to the algorithms, but it is sorted in this plot to ease understanding. This shows that NH performed better than expected and CI worse than expected.

When using a regression model to predict WRS_quiet using SSQ12 the best regression models achieved a root mean squared error (RMSE) of 9. Given that the target variable ranges from 0 to 100, this corresponds to a normalized RMSE (NRMSE) of 9% when normalized by the range of possible values. When inspecting shapely importance values, the most relevant predictors were questions one, nine and 10 in almost all the top models.

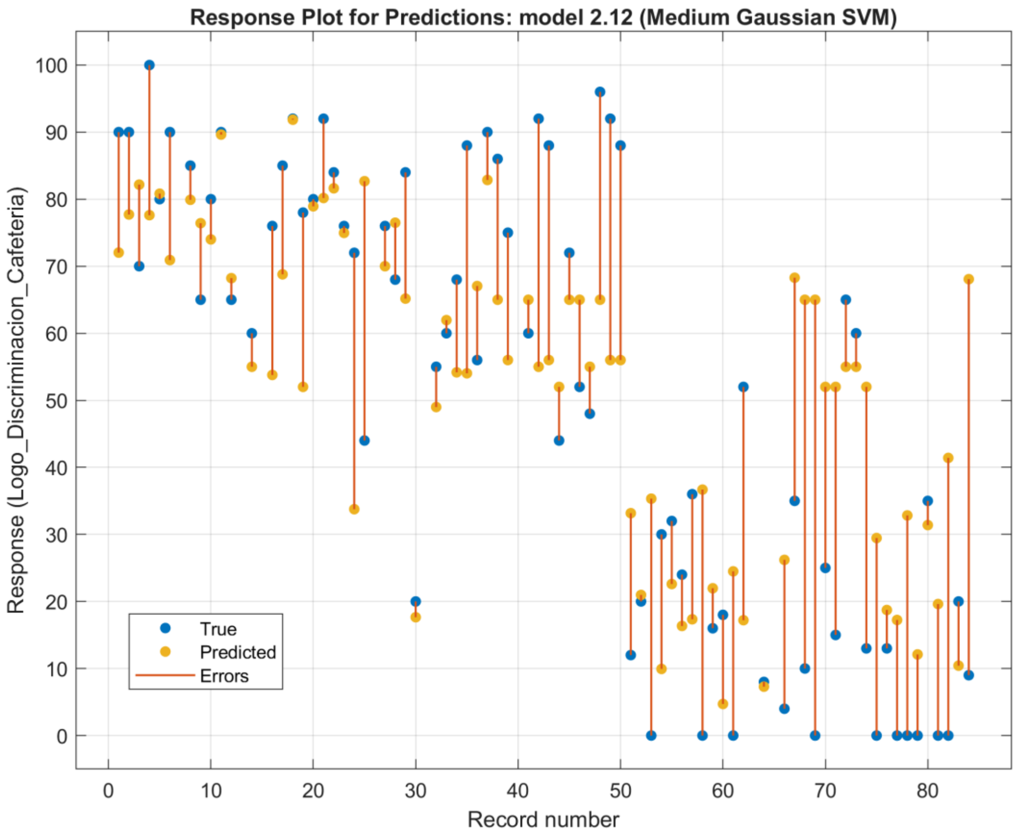

Figure S6. Response plot for model 12 (SVM). The plot appears much noisier than the previous case.

When using a regression model to predict WRS_cafe using SSQ12, the best models achieved a RMSE of 23. This scenario is more challenging than the previous one since the noise is very complex, and even users considered NH sometimes obtain poor results. Again, the best models tend to consider the ninth and tenth questions the most relevant ones, followed by the seventh.

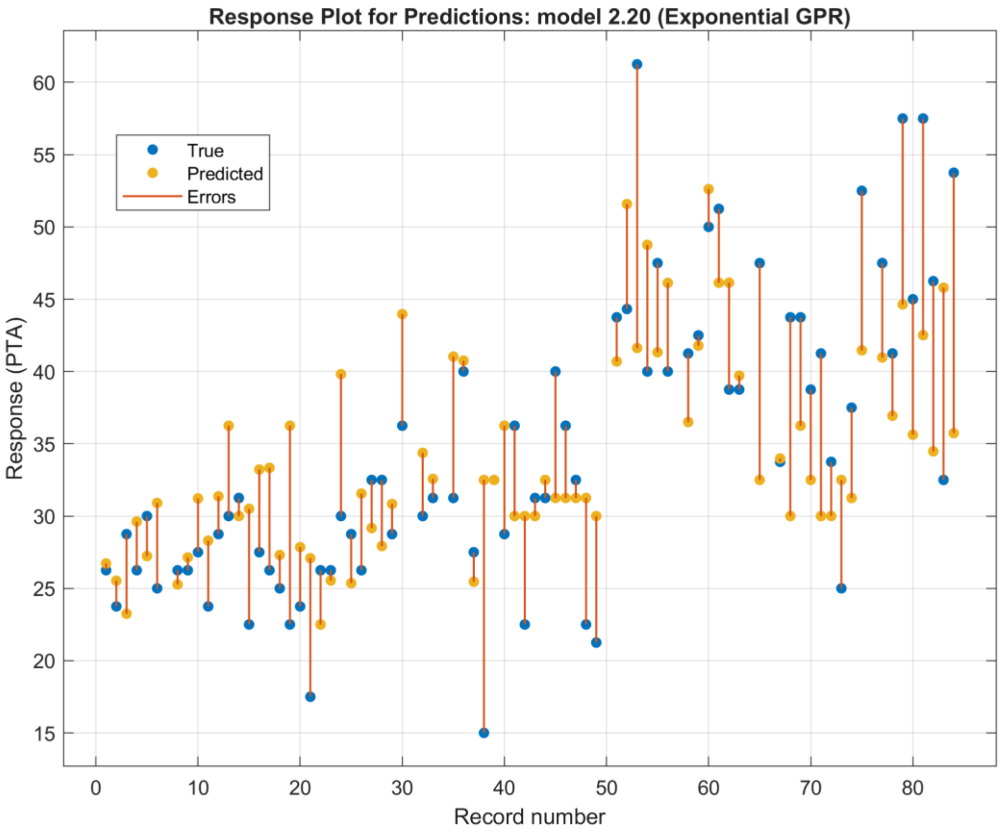

Figure S7. PTA values for each observation are shown as blue dots, predictions as yellow dots, and the measured error as orange lines. This figure illustrates that normal-hearing (NH) participants performed better than expected, while cochlear implant (CI) users performed worse than expected. This outcome is reasonable, given that all data were randomly mixed across trials.

When trying to estimate the value of the PTA using the SSQ12 questionnaire we obtained an RMSE of ~7. This result shows a certain separation between CI and NH groups, as spect, NH have a lower PTA than CI users. Given that the PTA spans a range of 0 to 120 dB HL, the NRMSE corresponds to approximately 6% of the full scale. In this case the most relevant predictors for the best models were again questions nine, ten, seven and six.
